# Factors associated with opioid overdose during medication-assisted treatment: How can we identify individuals at risk?

**DOI:** 10.1186/s12954-021-00521-4

**Published:** 2021-07-08

**Authors:** Vivian Y. O. Au, Tea Rosic, Nitika Sanger, Alannah Hillmer, Caroul Chawar, Andrew Worster, David C. Marsh, Lehana Thabane, Zainab Samaan

**Affiliations:** 1 Michael G. DeGroote School of Medicine, McMaster University, Hamilton, ON Canada; 2 Department of Psychiatry and Behavioral Neurosciences, McMaster University, 100 West 5th St, Hamilton, ON L8N 3K7 Canada; 3 Department of Health Research Methods, Evidence, and Impact, McMaster University, Hamilton, ON Canada; 4 Medical Sciences Graduate Program, McMaster University, Hamilton, ON Canada; 5 Neuroscience Graduate Program, McMaster University, Hamilton, ON Canada; 6 Department of Medicine, McMaster University, Hamilton, ON Canada; 7 Northern Ontario School of Medicine, Sudbury, ON Canada; 8 Canadian Addiction Treatment Centres, Markham, ON Canada; 9 ICES North, Sudbury, ON Canada; 10 Biostatistics Unit, Research Institute At St Joseph’s Healthcare, Hamilton, ON Canada; 11 Population Genomics Program, McMaster University, Hamilton, ON Canada

**Keywords:** Medication-assisted treatment, Opioid use disorder, Prospective observational study, Canada

## Abstract

**Background:**

Due to the loss of tolerance to opioids during medication-assisted treatment (MAT), this period may represent a time of heightened risk for overdose. Identifying factors associated with increased risk of overdose during treatment is therefore paramount to improving outcomes. We aimed to determine the prevalence of opioid overdoses in patients receiving MAT. Additionally, we explored factors associated with opioid overdose during MAT and the association between length of time enrolled in MAT and overdose.

**Methods:**

Data were collected prospectively from 2360 participants receiving outpatient MAT in Ontario, Canada. Participants were divided into three groups by overdose status: no history of overdose, any lifetime history of overdose, and emergency department visit for opioid overdose in the last year. We used a multivariate multinomial regression model to assess demographic and clinical factors associated with overdose status.

**Results:**

Twenty-four percent of participants reported a lifetime history of overdose (*n* = 562), and 8% reported an emergency department (ED) visit for opioid overdose in the last year (*n* = 179). Individuals with a recent ED visit for opioid overdose were in treatment for shorter duration (odds ratio [OR] 0.92, 95% confidence interval [CI] 0.87, 0.97, *p* = 0.001). Individuals with a lifetime or recent history of overdose were more likely to be younger in age (OR 0.93, 95% CI 0.89, 0.98, *p* = 0.007 and OR 0.84, 95% CI 0.77, 0.92, *p* < 0.001, respectively), report more physical symptoms (OR 1.02, 95% CI 1.01, 1.03, *p* = 0.005 and OR 1.03, 95% CI 1.01, 1.05, *p* = 0.005, respectively), and had higher rates of non-prescription benzodiazepine use (OR 1.87, 95% CI 1.32, 2.66, *p* < 0.001 and OR 2.34, 95% CI 1.43, 3.81, *p* = 0.001, respectively) compared to individuals with no history of overdose.

**Conclusions:**

A considerable number of patients enrolled in MAT have experienced overdose. Our study highlights that there are identifiable factors associated with a patient’s overdose status that may represent areas for intervention. In particular, longer duration in MAT is associated with a decreased risk of overdose.

## Background

Opioid overdose and overdose deaths remain a critical public health problem across North America. In 2018, nearly 70% of all drug overdose deaths involved an opioid and approximately 128 individuals die each day from opioid overdose in the USA [1]. However, these alarming statistics may not reflect the full extent of the opioid crisis. A recent study found that approximately 72% of unclassified drug overdoses between 1999 and 2016 involved opioids, suggesting that the number of opioid overdoses is undercounted [2].

For patients with opioid use disorder (OUD), medication-assisted treatment (MAT) with opioid agonists such as methadone and buprenorphine is effective in overdose prevention, decreasing rates of relapse, decreasing transmission of blood-borne infections, promoting retention in treatment, reducing use of illicit drugs, and improving employment and family functioning [3–5] . Most importantly, a number of studies have found a reduction in opioid-related mortality following MAT involving either methadone and buprenorphine by 59% and 38%, respectively [3–6] . Other studies have also demonstrated that individuals who were not receiving MAT had 8.1 times higher risk of overdose mortality when compared to those receiving MAT [4, 5].

Unfortunately, there is an ongoing “treatment gap” in which many individuals with OUD do not receive MAT [4, 6]. Even after opioid overdose, only 30% of patients are offered any medication for OUD [6]. Accordingly, strategies to improve initiation of MAT in individuals with OUD are certainly invaluable. Furthermore, there are some patients who, despite being treated with MAT, continue to have a risk of opioid overdose. Accordingly, many risk factors for overdose and mortality in individuals with OUD, with or without MAT, have been examined. For example, suicidal ideation [7, 8], younger age [9], male sex [5, 10], recent non-fatal overdose, type of MAT [5], shorter retention in MAT [5], recently leaving MAT[11], use of other central nervous system depressants, as well as comorbid physical conditions such as HIV [12] and mental health conditions such as depression and anxiety [12, 13] have all been identified as risk factors for overdose and mortality in individuals with OUD [4, 11].

Although MAT is effective in reducing overall risk of overdose, MAT may also represent a unique period of time during which certain patients who are receiving treatment may be at higher risk of overdose. In particular, individuals in the induction phase of MAT or treatment programs that require detoxification have a higher risk of overdose due to loss of opioid tolerance [3, 4] . With this consideration, factors that otherwise increase the risk of overdose in individuals with OUD but are not enrolled in treatment may not be generalizable to individuals actively receiving MAT. While most studies do examine differences in mortality rates and causes of death before, during, and after MAT treatment, they aim to capture overdose deaths as a measurement of the outcome of MAT without further elaboration or analysis on the clinical characteristics of these individuals stratified by overdose status [5]. Furthermore, few studies aim to describe the characteristics of individuals who are at risk of experiencing both fatal and non-fatal overdose during MAT beyond sex, HIV status, as well as the type of MAT [5, 12].

Understanding risk factors for any overdose, particularly during treatment, is critical and could allow clinicians to better identify and support individuals at higher risk of overdose and, consequently, mortality. Using data from a large cohort study of 2360 patients receiving MAT, we aim to better understand the prevalence of opioid overdose and factors associated with opioid overdose during MAT. Specifically, the objectives of this study are to:

1. Identify the lifetime prevalence of self-reported opioid overdoses among patients currently receiving MAT, and the past-year prevalence of self-reported overdose requiring care in the emergency department;
2. Explore factors associated with opioid overdoses during MAT;
3. Examine the association between length of time in MAT and opioid overdose.

## Methods

### Data

As part of the Pharmacogenetics of Opioid Substitute Treatment Response (POST) study, prospective observational data were collected from 2360 participants. Using a convenience sampling strategy with no stratification parameters, recruitment began in May 2018 from 31 outpatient MAT clinics located across Ontario, Canada. Patients attending regularly scheduled clinical appointments were consecutively approached for study participation and recruited voluntarily. As all clinical sites are run by central management team through the Canadian Addiction Treatment Centres (CATC), participants all received similar treatment protocols. These clinics provide services to more than 15,000 patients. MAT included either methadone or buprenorphine–naloxone, as buprenorphine as a monoproduct is not indicated for OUD treatment in Canada except in individuals who are pregnant [14]. Study inclusion criteria required participants to have a diagnosis of OUD as per the Diagnostic and Statistical Manual of Mental Disorders, 5th Edition (DSM-5), and to be receiving MAT for any length of time [15]. At study entry, all participants engaged in face-to-face interviews with trained interviewers and data were entered into the Research Electronic Data Capture tool [16, 17].

### Study instruments and measures

Information concerning demographical and substance use characteristics, clinical information on MAT treatment (including dose and duration) as well as physical and mental health symptoms were collected at study entry. Individuals were asked to self-report a lifetime history of opioid overdoses as well as any past-year history of opioid overdoses requiring emergency department care. To determine the prevalence of both psychological and physical symptoms, all participants completed the Maudsley Addiction Profile (MAP), which is an interviewer administered questionnaire measuring substance use, health risk behavior, physical and psychological health, and personal/social functioning in the last 30 days [18]. The 10-item physical health symptom scale encompasses five functional systems, including general symptoms including poor appetite and fatigue as well as cardiorespiratory, gastrointestinal, neurological, and musculoskeletal symptoms. Participants were asked to rate the frequency of experiencing each symptom on a five-point Likert-type scale using the expression “never,” “rarely,” “sometimes,” “often,” and “always” which corresponded to scores of 0–4, respectively. Psychological symptoms related to anxiety and depression were similarly measured and rated, but included symptoms such as tension, fear, nervousness, panic, hopelessness, worthlessness, anhedonia, loneliness, and suicidal ideation. Individual total symptom scores for the physical and psychological domains were obtained by adding the item scores for symptoms in each domain to produce a final score that ranged from 0 to 40.

Additionally, urine drug screen results were collected for every participant. Baseline urine drug screen results were obtained from samples collected for up to 12 months preceding study entry as per clinic treatment protocol. The FaStep Assay (Trimedic Supply Network Ltd., Concord, Ontario, Canada) was utilized to detect the presence of substances, including opioids and non-opioid substances [19]. An opioid-positive urine drug screen was defined as the detection of opioids other than methadone or buprenorphine. Results were used to calculate each patient’s percentage of positive urine drug screens for each substance.

### Analysis

All analyses were conducted using STATA Version 16.1 (StataCorp LP, College Station, TX, USA). Our first objective was to determine the prevalence of opioid overdoses in our study cohort. Patients were divided into 3 mutually exclusive groups based on their self-reported opioid overdose history, which included: (1) no reported overdoses, (2) any lifetime history of opioid overdoses, and (3) history of opioid overdose requiring emergency department (ED) care in the past year. Participants with both a lifetime history of overdose as well as an ED visit in the past year for opioid overdose were only included in the latter group to avoid duplication. Demographic and clinical data are presented using descriptive statistics based on group status, where continuous variables were summarized as means and standard deviations (SD) for normally distributed data or as a median with an interquartile range (IQR) for skewed data. All categorical variables were summarized using frequencies and percentages.

Our second and third objectives were to explore factors associated with opioid overdose during MAT as well as to examine the association between length of time in MAT and overdose. We initially constructed a univariate multinomial analysis using the patient’s opioid overdose status (no reported overdoses, lifetime history of overdoses, ED visit for overdose in the last year) as the dependent variable. This method was utilized to determine which covariates were associated with opioid overdose in an unadjusted analysis. Subsequently, a multivariate multinomial regression model was constructed using the same variables. Covariates including age [9], sex [10], MAT type [4–6], dose of MAT [4–6], years in treatment [4, 11], access and use of naloxone [3, 4], psychological symptoms of depression and anxiety [4, 11, 20], physical symptoms [4, 11], opioid abstinence at baseline [21], and benzodiazepine use [20] were all selected based on previous literature that suggested potential association with opioid overdose. The psychological symptoms score on MAP was not included in the regression model for a number of reasons. Firstly, the psychological symptom score includes suicidal ideation and it would have been redundant to include both in the analysis. Additionally, suicidal ideation is a more actionable symptom for clinicians to assess in patients, thus offering greater clinical utility when compared to a MAP score. Two variables were used to represent benzodiazepine use, including prescription and non-prescription use. Benzodiazepine use was defined as prescription use if patients had a prescription on their medical file for a benzodiazepine medication, while non-prescription use was defined by the presence of a benzodiazepine-positive urine drug screen and the absence of a prescription for a benzodiazepine medication on file. Benzodiazepine use was differentiated as such due to previous literature suggesting that non-prescribed benzodiazepines are associated with treatment discontinuation in MAT, while prescription benzodiazepines do not have such an impact [20]. All results from the regression model are reported as odds ratio (OR) with a 95% confidence interval (CI). The study reporting is according to the Strengthening the Reporting of Observational Studies in Epidemiology (STROBE) recommendations [22] [see Additional file 1].

## Results

Of the 2360 participants who were included in the analyses, 69% reported no history of opioid overdoses (*n* = 1619), 24% reported a lifetime history of opioid overdose (*n* = 562), and 8% reported history of an ED visit for opioid overdose in the last year (*n* = 179).

In Table 1, we present the demographic and clinical characteristics of participants by overdose history. Individuals with an ED visit for overdose in the last year were generally younger, with a mean age of 35 years (SD = 10) compared to a mean age of 39 years (SD = 11) in all participants. The highest rates of unemployment were seen in participants with an ED visit for overdose in the last year; only 17% of individuals were employed.

**Table 1:** Baseline demographic and clinical characteristics, by opioid overdose history (*N* = 2360)

Characteristic	Total sample *N* = 2360	No reported overdoses *n* = 1619 (68.6%)	Lifetime history of overdoses *n* = 562 (23.8%)	ED visit for overdose in the last year *n* = 179 (7.6%)
**Sociodemographic**				
Age; mean (SD)	39.3 (10.9)	40.0 (10.8)	38.8 (11.1)	34.9(9.6)
Female sex^a^; *n* (%)	1046 (44.3%)	728 (45%)	245 (43.7%)	73(40.8%)
Ethnicity; *n* (%) Caucasian Other	1705 (72.3%) 655 (27.8%)	1154 (71.3%) 465 (28.7%)	422 (75.1%) 140 (24.9%)	129 (72.1%) 50 (27.9%) [3]
Married or common law;* n* (%)	691 (29.3%)	515 (31.8%)	135 (24%)	41 (22.9%)
High school education; *n* (%)	668 (28.3%)	473 (29.2%)	153 (27.2%)	42 (23.5%)
Children; *n* (%)	1615 (68.4%)	1132 (69.9%)	366 (65.1%)	117 (65.4%)
Currently working; *n* (%)	780(33.1%)	592 (36.6%)	158 (28.1%)	30 (16.8%)
Receiving social assistance^b^; *n* (%)	1298 (55%)	827 (51.1%)	350 (62.3%)	121 (67.6%)
**Treatment**				
Type of MAT^c^; *n* (%) Methadone Suboxone	1868 (79.3%) 488 (20.7%)	1270 (78.6%) 346 (21.4%)	456 (81.3%) 105 (18.7%)	142 (79.3%) 37 (20.7%)
Dose; mean (SD) Methadone Suboxone	70.5 (40.6) 12.0 (6.8)	69.5(42.4) 11.9 (6.9)	76.9(37.1) 12.6 (6.4)	58.9 (31.5) 11.2 (6.4)
Years in treatment; median (Q1, Q3)	2.6 (0.8, 6)	3(1, 6.75)	2.4 (0.8, 7)	0.6(0.2,2)
Previous Treatment for Opioid Dependence^a^; *n* (%)	804 (34.1%)	500 (30.9%)	217 (38.6%)	87 (48.6%)
Abstinent from opioid use at study entry^d^; *n*(%)	732 (31.1%)	537 (33.3%)	166 (29.7%)	29 (16.3%)
Percentage of opioid-positive urine drug screens if non-abstinent at study entry^d^; mean (SD)	16.3 (23.3)	15.1 (22.9)	16.7 (22.8)	25.9 (25.9)
**Substance use**				
Age start using opioids regularly^a^; mean (SD)	24.9 (9.3)	25.9 (9.6)	22.7 (8.3)	22.4 (8.1)
IV drug use (past 30 days); *n*(%)	375 (15.9%)	164 (10.1%)	127 (22.6%)	84 (46.9%)
Alcohol use (past 30 days)^e^; *n* (%)	870 (36.9%)	588 (36.3%)	208 (37%)	74 (41.6%)
Prescription benzodiazepine use, *n* (%)	369 (15.6%)	242 (15%)	103 (18.3%)	24 (13.4%)
Non-prescription use of benzodiazepines (past 30 days)^e^; *n* (%)	180 (7.6%)	88 (5.4%)	64 (11.4%)	28 (15.7%)
Cannabis use (past 30 days)^e^; *n* (%)	1257 (53.3%)	846 (52.3%)	301 (53.6%)	110 (61.8%)
Have access to naloxone kits; *n* (%)	1840 (78%)	1210 (74.7%)	472 (84%)	158 (88.3%)
Know how to use naloxone; *n* (%)	1907 (80.8%)	1243 (76.8%)	493 (87.7%)	171 (95.5%)
**Mental and physical health**				
Suicidal ideation; *n *(%)	489 (20.7%)	298 (18.4%)	134 (23.8%)	57 (31.8%)
Total physical symptoms score on MAP^c^; mean (SD)	14.3 (8.0)	13.8 (7.9)	15.2 (8.0)	16.0 (8.5)
Total psychological symptoms score on MAP^f^; mean (SD)	11.7 (9.0)	10.7 (8.8)	13.4(8.9)	14.6 (9.5)

SD = Standard deviations, MAT =  medication-assisted treatment, IV = intravenous, MAP = Maudsley Addiction Profile

^a^Data available for 2359 participants

^b^Data available for 1580 participants

^c^Data available for 2356 participants

^d^Data available for 2352 participants

^e^Data available for 2358 participants

^f^Data available for 2354 participants

With respect to treatment characteristics, 79% of patients were receiving methadone, while the remainder received buprenorphine–naloxone. This ratio is similar in all groups. Participants who had an ED visit for overdose in the last year were receiving lower mean doses of both methadone (59 mg/day, SD = 32) and buprenorphine–naloxone (11 mg/day, SD = 6) when compared to both of the other groups. Participants with no reported overdoses were receiving on average 70 mg of methadone/day or 12 mg of buprenorphine–naloxone/day, while those with a history of overdoses were receiving around 77 mg of methadone/day and 13 mg of buprenorphine–naloxone/day. Additionally, participants with an ED visit for overdose were receiving treatment for a median of 0.6 years (IQR = 0.2, 2), which is a shorter length of time when compared to both of the other groups, who had spent a median length of 3 and 2.4 years, respectively, in MAT.

Examining substance use characteristics, participants with an ED visit for overdose in the last year had the highest rates of IV drug use at 47% and only 16% of individuals were abstinent from opioid use at baseline. Rates of alcohol and cannabis use were similar across all groups. However, participants who had an ED visit for overdose in the last year had the highest rate of non-prescription use of benzodiazepines, with 16% reporting use in the last 30 days. Additionally, this group also had the lowest rates of prescription benzodiazepine use. Of note, access to naloxone kits and knowledge on using naloxone were both highest in participants who had an ED visit in the last year for overdose. Lastly, individuals with an ED visit for overdose initiated opioid usage at an average age of 24 years compared to 26 and 23 years in the other groups. 

Regarding physical and mental health, 32% of individuals with an ED visit for overdose reported suicidal ideation compared to 18% in individuals with no reported overdoes and 24% in those with a lifetime history of overdoses. Lastly, individuals with an ED visit for overdose also had higher total scores on both the physical and psychological components of the MAP.

In Table 2, we present the results of our multinomial regression analysis, identifying factors associated with overdose history. Adjusting for other demographic and clinical factors, both participants with a history of overdose and those with an ED visit for overdose in the last year were younger in age when compared to the group with no reported overdoses (OR 0.93, 95% CI 0.89, 0.98, *p* = 0.007 and OR 0.84, 95% CI 0.77, 0.92, *p* < 0.001, respectively). Compared to the group with no reported overdoses, those with an ED visit in the last year have spent less time in treatment (OR 0.92, 95% CI 0.87, 0.97, *p* = 0.001). Both those with a history of overdose and those with an ED visit for overdose in the last year were more likely to report access to naloxone kits compared to the group with no reported overdoses (OR 1.59, 95% CI 1.23, 2.06, *p* < 0.001 and OR 1.90, 95% CI 1.17, 3.08, *p* = 0.01, respectively). Additionally, both groups were reporting higher numbers of physical symptoms on the MAP (OR 1.02, 95% CI 1.01, 1.03, *p* = 0.005 and OR 1.03, 95% CI 1.01, 1.05, *p* = 0.005, respectively) as well as higher rates of non-prescription use of benzodiazepines (OR 1.87, 95% CI 1.32, 2.66, *p* < 0.001 and OR 2.34, 95% CI 1.43, 3.81, *p* = 0.001, respectively). Patients with an ED visit for overdose in the last year were less likely to be abstinent from opioids at study entry (OR 0.49, 95% CI 0.32, 0.75, *p* = 0.001). Adjusting for other covariates, we did not identify a statistically significant association between overdose status and suicidal ideation for both groups with a history of overdose as well as those with an ED visit for overdose in the last year (OR 1.17, 95% CI 0.92, 1.50, *p* = 0.202 and OR 1.40, 95% CI 0.97, 2.03, *p* = 0.071, respectively). Lastly, no association was found between overdose status and sex, type of MAT, alcohol use in the past 30 days as well as use of prescription benzodiazepines.

**Table 2:** Univariate and multivariate multinomial analysis of demographic and clinical factors associated with overdose status (N = 2360)

		Univariate multinomial analysis			Multivariate multinomial analysis		
Overdose status	Covariate	OR	95% CI	*p* value	OR	95% CI	*p* value
No reported overdoses [reference]	–	–	–	–	–	–	–
Lifetime history of overdoses	Age by five-year increments	0.99	0.98, 1.00	0.024	0.93	0.89, 0.98	0.007
	Sex	0.95	0.78, 1.15	0.595	0.88	0.72, 1.08	0.210
	MAT	0.85	0.66, 1.08	0.175	1.19	0.88, 1.60	0.254
	Dose	1.00	1.00, 1.01	< 0.001	1.00	1.00, 1.01	0.005
	Years in treatment	1.02	1.00, 1.04	0.061	1.02	1.00, 1.04	0.082
	Have access to naloxone kits	1.77	1.38, 2.28	< 0.001	1.59	1.23, 2.06	< 0.001
	Suicidal Ideation	1.39	1.10, 1.75	0.005	1.17	0.92, 1.50	0.202
	Total physical symptoms score on MAP	1.02	1.01, 1.04	< 0.001	1.02	1.01, 1.03	0.005
	Alcohol use (past 30 days)	1.03	0.84, 1.26	0.776	1.01	0.82, 1.24	0.927
	Prescription benzodiazepine use	1.28	0.99, 1.65	0.059	1.08	0.83, 1.42	0.559
	Non-prescription use of benzodiazepines (past 30 days)	2.23	1.59, 3.13	< 0.001	1.87	1.32, 2.66	< 0.001
	Opioid abstinence at study entry	0.85	0.69, 1.04	0.122	0.92	0.74, 1.14	0.445
ED visit for overdose in the last year	Age by 5-year increments	0.95	0.94, 0.97	< 0.001	0.84	0.77, 0.92	< 0.001
	Sex	0.84	0.62, 1.15	0.286	0.77	0.55, 1.08	0.128
	MAT	0.96	0.65, 1.40	0.819	0.73	0.45, 1.16	0.181
	Dose	1.00	0.99, 1.00	0.015	1.00	0.99, 1.00	0.068
	Years in treatment	0.88	0.84, 0.92	< 0.001	0.92	0.87, 0.97	0.001
	Have access to naloxone kits	2.54	1.59, 4.06	< 0.001	1.90	1.17, 3.08	0.01
	Suicidal Ideation	2.07	1.48, 2.91	< 0.001	1.40	0.97, 2.03	0.071
	Total physical symptoms score on MAP	1.03	1.02, 1.05	< 0.001	1.03	1.01, 1.05	0.005
	Alcohol use (past 30 days)	1.25	0.91, 1.71	0.170	1.02	0.73, 1.43	0.886
	Prescription benzodiazepine use	0.88	0.56, 1.38	0.582	1.02	0.63, 1.63	0.944
	Non-prescription use of benzodiazepines (past 30 days)	3.25	2.05, 5.13	< 0.001	2.34	1.43, 3.81	0.001
	Opioid abstinence at study entry	0.39	0.26, 0.59	< 0.001	0.49	0.32, 0.75	0.001

*OR* Relative risk ratio, *CI* confidence interval

Due to the discrepancies in the univariate and multivariate regression models, we conducted a post hoc analysis to identify possible associations between suicidal ideation and the other covariates (Table 3). Factors found to be associated with increased odds of reporting suicidal ideation included younger age, shorter time in treatment, increased physical symptoms, alcohol use, prescription and non-prescription benzodiazepine use, and ongoing opioid use at study entry (Table 3). Individuals reporting suicidal ideation were most likely to use prescription or non-prescription benzodiazepines (OR 1.69, 95% CI 1.32, 2.18, *p* < 0.001 and OR 2.35, 95% CI 1.70, 3.25, *p* < 0.001, respectively).

**Table 3:** Multivariable model of factors associated with suicidal ideation

Covariate	OR	95% CI	*p*
Age	0.98	0.97, 0.99	< 0.001
Sex	0.91	0.74, 1.11	0.34
MAT	1.02	0.80, 1.31	0.85
Dose	1.00	1.00, 1.00	0.24
Years in Treatment	0.97	0.95, 1.00	0.02
Have access to naloxone kits	1.60	1.23, 2.08	< 0.001
Total physical symptoms score on MAP	1.08	1.06, 1.09	< 0.001
Alcohol Use (past 30 days)	1.21	0.99, 1.48	0.07
Prescription benzodiazepine use	1.69	1.32, 2.18	< 0.001
Non-prescription use of benzodiazepines (past 30 days)	2.35	1.70, 3.25	< 0.001
Opioid abstinence at baseline	0.68	0.54, 0.85	0.001

*OR* Odds ratio, *CI* confidence interval, *MAT* medication-assisted treatment, *MAP* Maudsley Addiction Profile

## Discussion

Among this cohort of patients receiving MAT, approximately 8% had an ED visit for an opioid overdose in the last year, while 24% reported a lifetime history of overdoses despite being on treatment. Therefore, for a notable number of patients who have been diagnosed with OUD and are receiving MAT, factors that contribute to overdose during MAT are important to consider.

The characteristics we found to be associated with lifetime and past-year history of opioid overdose spanned across sociodemographic and treatment factors, as well as polysubstance use and comorbidity.

### Sociodemographic characteristics

Patients who had a history of overdose or an ED visit for overdose in the last year were found to be significantly younger in age when compared to the group with no reported overdoses. This finding is in keeping with current research that younger individuals are often at higher risk of both fatal and non-fatal overdoses, highlighting the need for further advocacy and research to improve outcomes for this population [9, 21, 23]. While OUD can impact any individual regardless of age, the pediatric and adolescent stages represent important periods for intervention as most individuals report use before 25 years of age [9, 24, 25]. This finding is similarly reflected in our data, where patients who had an ED visit for opioid overdose in the last year started using opioids at a mean age of 22.4 years. Recent literature has highlighted that a large proportion of individuals in treatment for OUD are actually young adults but only a small number of them are receiving MAT [24–26]. This underuse of an effective treatment in a vulnerable population is certainly alarming [27]. For these reasons, further research is needed on identifying barriers to accessing treatment both within and outside the clinical environment for younger adults with OUD [24, 26].

### Treatment characteristics

Patients who had an ED visit for opioid overdose in the last year had spent fewer years in treatment in comparison to patients who had no reported overdoses, with a median of 0.6 years. This finding is consistent with current literature that suggests that better retention in MAT is associated with reduced overdose mortality [5, 11]. Additionally, the group with ED visits for overdose in the last year also had a significantly greater percentage of opioid-positive urine drug screens upon study entry. This finding, in parallel with the younger mean age of this group, is consistent with current literature for risk factors predictive of discontinuing MAT prematurely [21]. More research into strategies for maintaining engagement with MAT in populations at higher risk of discontinuation may have important benefits for preventing overdose.

### Substance use characteristics

More than 88% of patients who had an ED visit for overdose in the last year reported access to naloxone kits, which is significantly higher compared to the group with no reported overdoses where only 75% of patients had access to naloxone. These numbers are in keeping with the nationwide efforts to increase naloxone access to individuals at risk of experiencing or witnessing an overdose event [28, 29]. Although it is encouraging that individuals at highest risk also have the greatest access to naloxone, these numbers are still concerning. Naloxone has been demonstrated to be a cost-effective agent for treating opioid overdose as well as reducing opioid overdose deaths [30]. Current literature suggests that all individuals who use opioids, who have a history of substance use disorder or are in close contact with those who do use opioids should have access to a naloxone kit [3, 4, 28, 30–33]. Practically, this suggests that all individuals who are enrolled in MAT should have access to a naloxone kit as they fulfill all of the criteria [3, 4, 28, 30–33]. Accordingly, physicians have long advocated for this change, with the US Surgeon General releasing a public health advisory in 2018 advocating for increased naloxone access [34]. Additionally, many patients engaged in MAT are willing to carry and use naloxone kits and those who have experience with naloxone believe it to be both effective and necessary [28, 35]. With support from the literature, prescribers, as well as patients, it seems that continuing to improve access to naloxone for any individual at risk should be considered. Currently, many researchers have identified the potential legislative and practical barriers surrounding the access, administration, and distribution of naloxone. On the legislative level, despite all 50 states passing laws to improve naloxone access to the public, there remains significant variation in these laws as well as prevalent underuse of naloxone [28]. Additionally, Good Samaritan laws have only been established in 40 states and there are significant differences in the immunity provided to individuals who report overdoses [28]. In comparison, 8 of 13 provinces and territories in Canada have created initiatives for take-home naloxone program and, as a country, have been working to improving access by expanding the list of medical professionals able to provide and administer prescriptions of naloxone, improving rules for eligibility, as well as changing naloxone’s prescription-only status [36]. Regarding practical considerations, providing training on naloxone use, the physical size of the kit, as well as promotion from both the media and prescribers is needed to increase awareness regarding the utility of naloxone [35]. Future studies should focus on identifying barriers for improving access to naloxone for all individuals at risk, including those who are enrolled in MAT but have no reported overdoses.

With regards to additional substance use characteristics, the non-prescription use of benzodiazepines was more likely in both patients with a history of overdose as well as those with ED visits for overdose in the last year. There was no significant association between prescription benzodiazepine use and overdose status. These findings are important for a number of different reasons. Firstly, this finding highlights the notable differences between use of prescription versus non-prescription benzodiazepines. Multiple studies have found that the use of prescription benzodiazepines during opioid treatment does not impact retention or outcomes when compared with non-prescription use of benzodiazepines [20, 37]. Similarly, the use of non-prescription benzodiazepines was associated with discontinuation of MAT [20]. Our findings are in keeping with the current literature, but more research is required to elucidate the difference between individuals who use prescribed or non-prescribed benzodiazepines. Additionally, these findings should not be interpreted as advocacy for the indiscriminate prescription of benzodiazepines during MAT. Interactions between opioids and benzodiazepines may increase the likelihood of death due to respiratory depression [38]. Furthermore, while studies found no effect of prescription benzodiazepine on MAT treatment, they also demonstrated a concomitant increase in the likelihood of accidental injuries, ED visits, as well as issues with physical dependence [20, 37]. Studies solely examining the impact of benzodiazepine use without separate examination of prescription versus non-prescription populations have also found increases in risk of drug overdose [39, 40]. Combined with findings that benzodiazepines are frequently co-prescribed with MAT, more research is needed to provide both prescriber and patient education on safe use of benzodiazepines with MAT [20, 41, 42].

### Physical health characteristics

Both patients who had a history of overdose as well as those with an ED visit for overdose in the last year reported more physical symptoms on the MAP compared to the group with no reported overdoses. Overdose events are difficult to classify by intent, but it stands to reason that a proportion of overdoses may be associated with suicidal thoughts [7, 43, 44]. While there is limited literature examining the association between physical symptoms and suicidal ideation in individuals with OUD, multiple recent studies demonstrated that physical illness is associated with an increased risk of suicide, particularly in the presence of psychiatric comorbidities [44–48]. These findings suggest a need for further exploration into the associations between physical illness, overdose, and suicide risk among individuals with OUD in order to better support patients.

### Mental health characteristics

When adjusting for other demographic and clinical factors, we found no significant association between suicidal ideation and lifetime or past-year history of opioid overdose. This result was unexpected given the incremental increase in reports of suicidal ideation between the groups that correlated with experiences of overdose. Some researchers have recently begun to elucidate the differences between an opioid overdose and a suicide attempt. However, there is no current consensus on the difference between the two and there is a defined absence of attempts to integrate research on suicidal attempts and overdose in individuals with substance use disorders [7, 43, 44].

Our post hoc analysis to identify possible associations between suicidal ideation and the other covariates found that individuals reporting suicidal ideation were most likely to use prescription or non-prescription benzodiazepines. This is an alarming finding as benzodiazepines are often identified in opioid overdose deaths, and current literature suggests that benzodiazepine prescriptions in the general population are associated with increased risk for both attempting and completing suicide [49, 50]. However, few researchers have examined this relationship in individuals with SUD as well as the differences in impact between prescription and non-prescription benzodiazepines [49, 50]. Given the significant association between suicidal ideation and both prescription and non-prescription use of benzodiazepines, further exploration into the association is warranted in future research.

### Strengths and limitations

This study is strengthened by its large sample size as well as multisite enrollment, increasing confidence and producing more generalizable results. Additionally, the MAP, a validated research instrument used to assess treatment outcomes in individuals with SUD, was used to interview patients. As with all observational studies, no causal relationship can be concluded and the potential for unknown confounding variables exists. Additionally, although we control for length of time in our regression model, the exact timeline of when participants experienced an overdose cannot be identified. Therefore, it is possible that participants experienced the overdose identified in the interview before actually initiating MAT. Future studies that identify overdose events in relation to MAT initiation should be conducted to better elucidate the relationship. This study is also limited by healthy user and volunteer bias wherein patients who are more satisfied with treatment or experiencing less symptoms may volunteer compared to their peers. It is likely that individuals who carry the highest illness burden may be least likely to participate; for this study of patients self-reporting history of opioid overdoses, individuals who died by overdose would not be included. Furthermore, there is a risk of misallocation bias if participants underestimate their history of opioid overdose as the term “overdose” was not explicitly defined in the interview. Additionally, incidences where individuals required paramedic care but did not present to the emergency department are not described in the data. Future studies using administrative health records (e.g., hospital records, emergency medical services records, coroner reports) are required to better capture all individuals who may be affected by opioid overdose.

Finally, the generalizability of our results outside of Ontario, Canada, must be considered, particularly due to differences in the role of MAT as a harm-reduction treatment as compared to an abstinence-based treatment, in different jurisdictions. MAT protocols in Canada function on the basis of harm reduction, and therefore, individuals are not excluded from treatment based on ongoing opioid use, other substance use, or overdose history.

## Conclusions

Our findings indicate that a considerable proportion of patients enrolled in MAT are affected by past or recent overdose. Additionally, there are significant differences in sociodemographic, treatment, substance use, and mental/physical health characteristics among patients stratified by their overdose status. Our study’s findings also concur with previous literature that suggests better retention in MAT is associated with a decreased likelihood of overdose [5, 11]. These findings are significant as while MAT reduces overall mortality in these individuals, it also represents a period of increased risk in the event of an overdose due to potential loss of tolerance to opioids [3, 4]. Therefore, future research to incorporate both the identification and consideration of clinical characteristics that may indicate higher risk for overdose into MAT treatment programs may serve to further improve outcomes. This study also highlights the need for further exploration into the accessibility of MAT across various age groups, potential barriers to naloxone access, the impact of physical illness on overdose and suicidal ideation, as well as differences in risk between the use of prescription and non-prescription benzodiazepines in both suicidal ideation and overdose during MAT.

## Data Availability

The datasets used and/or analyzed during the current study are available from the corresponding author on reasonable request.

## Abbreviations

CATC: Canadian Addiction Treatment Centres
CI: Confidence interval
DSM-5: Diagnostic and Statistical Manual of Mental Disorders, 5^th^ Edition
ED: Emergency department
IQR: Interquartile range
MAP: Maudsley Addiction Profile
MAT: Medication-assisted treatment
OR: Odds ratio
OUD: Opioid use disorder
POST: Pharmacogenetics of Opioid Substitute Treatment Response
SD: Standard deviations
STROBE: The Strengthening the Reporting of Observational Studies in Epidemiology
